# From Burnout to Resilience: Addressing Moral Injury in Nursing Through Organizational Innovation in the Post-Pandemic Era

**DOI:** 10.3390/healthcare13212822

**Published:** 2025-11-06

**Authors:** Enășoni Sorina, Dorin Novacescu, Alina Cristina Barb, Alexandru Ciolofan, Cristina Stefania Dumitru, Flavia Zara, Raul Patrascu, Alexandra Enache

**Affiliations:** 1 Doctoral School, Victor Babes University of Medicine and Pharmacy Timisoara, E. Murgu Square, No. 2, 300041 Timisoara, Romania; sorina.enasoni@umft.ro; 2 Department II of Microscopic Morphology, Discipline of Histology, Victor Babes University of Medicine and Pharmacy Timisoara, E. Murgu Square, No. 2, 300041 Timisoara, Romania; novacescu.dorin@umft.ro (D.N.); toma.alina@umft.ro (A.C.B.); flavia.zara@umft.ro (F.Z.); 3 Department IX, Discipline of Surgical Semiology I, Victor Babes University of Medicine and Pharmacy Timisoara, E. Murgu Square, No. 2, 300041 Timisoara, Romania; ciolofan.alexandru@umft.ro; 4 Department of Functional Sciences, Victor Babes University of Medicine and Pharmacy Timisoara, 300041 Timisoara, Romania; patrascu.raul@umft.ro; 5 Department VIII, Discipline of Forensic Medicine, Bioethics, Deontology and Medical Law, Victor Babes University of Medicine and Pharmacy Timisoara, E. Murgu Square, No. 2, 300041 Timisoara, Romania; enache.alexandra@umft.ro; 6 Center for Ethics in Human Genetic Identifications, Victor Babes University of Medicine and Pharmacy Timisoara, E. Murgu Square, No. 2, 300041 Timisoara, Romania

**Keywords:** nursing, burnout, moral injury, resilience, post-pandemic care, leadership, workload management, organizational innovation

## Abstract

The COVID-19 pandemic has profoundly amplified burnout and moral injury among nurses, exposing structural vulnerabilities in healthcare systems and accelerating workforce attrition. Beyond the acute crisis, nurses continue to face chronic staff shortages, overwhelming workloads, and unresolved ethical tensions that compromise both well-being and quality of care. Synthesis of recent meta-analyses in this review indicates that nurse burnout during the pandemic ranged between 30% and 50%, illustrating the magnitude of the problem. Particular attention is given to innovative organizational strategies that foster resilience, including workload redistribution, enhanced professional autonomy, supportive leadership, and the integration of digital technologies such as telecare. Comparative perspectives across healthcare systems illustrate how policy reforms, staffing models, and ethical frameworks can mitigate psychological distress and strengthen organizational resilience. By reframing burnout and moral injury not only as individual challenges but as systemic phenomena requiring structural solutions, this review emphasizes the imperative of multilevel interventions. Building resilient nursing workforces through innovation, leadership, and evidence-based policies is essential for sustaining high-quality patient care in the post-pandemic era.

## 1. Introduction

The COVID-19 pandemic has profoundly disrupted healthcare systems worldwide, exposing structural vulnerabilities and placing unprecedented strain on the nursing workforce. Nurses, positioned at the frontline of care delivery, faced overwhelming workloads, shortages of protective equipment, and ethically distressing decisions that intensified psychological distress and moral conflict. While these challenges predated the pandemic, they escalated under crisis conditions, producing long-term consequences for nurses’ well-being, patient safety, and organizational resilience. Both burnout and moral injury emerged as key indicators of this systemic strain; their conceptual distinctions are outlined in the following section [1,2,3]. Burnout, characterized by emotional exhaustion, depersonalization, and reduced professional efficacy, was already highly prevalent before 2020 but surged during the pandemic. Meta-analyses report that nearly one in two healthcare professionals experienced significant burnout symptoms during this period, with nurses being among the most affected groups [2,3]. At the same time, the concept of moral injury—initially developed in military contexts—gained traction in healthcare. It refers to the psychological distress that arises when professionals are forced to act against deeply held ethical values, such as rationing scarce resources or implementing restrictive policies, situations repeatedly encountered during COVID-19 [4,5]. Therefore, this review synthesizes current evidence on burnout and moral injury among nurses and addresses the existing gap in understanding how system-level innovations can foster resilience and workforce sustainability in the post-pandemic era.

Although much of the evidence originates from studies conducted during the pandemic’s acute phases (2020–2022), recent longitudinal analyses and umbrella reviews confirm that nurse burnout has remained elevated above pre-pandemic baselines through 2023–2025 [6]. Emotional exhaustion and intent-to-leave persist at clinically significant levels, particularly in high-acuity environments such as intensive care units (ICU) and emergency departments (ED), but also across general inpatient and long-term care settings [7,8]. This sustained trajectory from acute crisis to post-pandemic recovery highlights that chronic factors—such as workload, nurse-to-patient ratios, and organizational trust—are enduring drivers of burnout, rather than temporary disruptions. This framing situates the review within a post-pandemic continuum, using acute-phase data to expose systemic vulnerabilities that continue to impact the nursing workforce [9,10].

Emerging evidence indicates that both burnout and moral injury remain highly relevant in the post-pandemic era. Chronic staffing shortages, workload redistribution, and unresolved ethical tensions continue to fuel psychological distress among nurses. For example, a recent longitudinal study revealed that emotional exhaustion and moral distress persisted or even worsened among nurses beyond the acute phase of the pandemic, despite declining infection rates [11]. Furthermore, moral injury has been strongly correlated with post-traumatic stress disorder (PTSD), depression, anxiety, and suicidal ideation, underscoring its long-term implications for the mental health and retention of healthcare workers [5,12,13].

This article presents a narrative review of the literature on burnout and moral injury in nurses, with a particular focus on the post-pandemic context. Building on peer-reviewed evidence published in English between January 2020 and September 2025, the review critically examines prevalence, mechanisms, and consequences, while emphasizing innovative organizational strategies, leadership approaches, and technological solutions that can enhance resilience and sustainability in nursing care. Details of the search strategy, eligibility criteria, and thematic synthesis are provided in the dedicated Methods section. Although much of the evidence synthesized derives from studies conducted during the acute phases of the COVID-19 pandemic (2020–2022), these data offer essential insights into systemic vulnerabilities that persist in the post-pandemic recovery period. This continuity underscores that the challenges faced by nurses remain chronic and require sustained, system-level interventions.

## 2. Methods

This review follows the SANRA (Scale for the Assessment of Narrative Review Articles) quality criteria, as a narrative meta-synthesis was considered most appropriate given the conceptual heterogeneity of the studies and the integrative aim of the paper.

Literature was searched in PubMed/MEDLINE, Scopus, Web of Science, and Google Scholar for the period January 2020–September 2025, using Boolean combinations of: (nurse AND (“burnout” OR “moral injury” OR “moral distress”) AND (“COVID-19” OR “post-pandemic” OR “resilience”)) * and additional terms such as leadership, staffing, autonomy, and digital tools.

Inclusion criteria: peer-reviewed empirical studies, systematic reviews, and meta-analyses in English focusing on nurses or mixed healthcare worker samples reporting nurse-specific data on burnout, moral distress/injury, resilience, or organization-level interventions.

Exclusion criteria: editorials, commentaries, non-peer-reviewed sources, and non-clinical opinion pieces.

Titles/abstracts were screened manually, followed by full-text review of eligible records. Findings were synthesized thematically and conceptually across four analytical domains: (1) conceptual definitions and overlaps; (2) prevalence and severity; (3) determinants and theoretical models; and (4) organizational and policy interventions.

Given the heterogeneity in study designs and measurement tools, no quantitative meta-analysis was conducted; instead, we provide a critical narrative integration emphasizing convergent evidence, major achievements, and remaining research gaps.

## 3. Burnout in Nursing During COVID-19

### 3.1. Conceptual Background and Definition

Burnout in nursing is characterized by emotional exhaustion, depersonalization, and reduced professional accomplishment, dimensions operationalized most frequently through the Maslach Burnout Inventory (MBI) [14,15,16]. During the COVID-19 pandemic, these dimensions were exacerbated, as nurses confronted overwhelming patient care demands, shortages of personal protective equipment, and recurrent exposure to ethically distressing situations [1,17].

Recent studies have reframed burnout as not merely an individual psychological syndrome but also as a structural and organizational challenge. Evidence shows that nurse burnout is strongly linked to reduced patient safety, lower quality of care, and increased risk of medical errors [18,19]. Moreover, longitudinal analyses suggest that persistent burnout contributes directly to workforce attrition, thereby undermining the resilience of healthcare systems in the post-pandemic era [20]. This evolving conceptualization underscores the necessity of addressing burnout in nursing through a multidimensional lens—acknowledging it as both a mental health concern for individuals and a systemic vulnerability within healthcare delivery [21].

### 3.2. Prevalence and Epidemiology

Burnout among nurses during the COVID-19 pandemic has been extensively documented, with prevalence rates consistently exceeding pre-pandemic levels. Recent meta-analyses and large-scale surveys indicate that between 30% and 50% of nurses worldwide experienced clinically significant burnout symptoms, with notable variation by region, specialty, and pandemic phase [3,14,22]. These findings highlight the global yet context-dependent nature of nurse burnout, which is influenced by workload intensity, ethical conflict, and systemic resource gaps. To provide a clearer overview and avoid redundancy, the prevalence data reported across multiple meta-analyses and surveys were consolidated into a comparative summary table. Table 1 synthesizes the main findings by region and nursing specialty, highlighting variations in burnout prevalence and the contextual factors influencing these differences.

These comparative findings illustrate substantial variability across settings, with critical-care and emergency nurses showing the highest burnout levels. Structural factors such as staffing ratios, leadership support, and access to protective resources appear to moderate these outcomes, underscoring the systemic nature of burnout in nursing.

Beyond regional and specialty-related variability, burnout prevalence is also shaped by sociodemographic and organizational factors. Evidence from large-scale studies indicates that younger and less experienced nurses are disproportionately affected, partly due to heavier workloads, limited autonomy, and reduced institutional support [14,21]. Gender differences have likewise been documented, with female nurses reporting higher emotional exhaustion, whereas male nurses tend to show greater depersonalization [3,14]. These disparities highlight how demographic variables interact with systemic inequities rather than acting independently. Addressing such imbalances requires organizational strategies, including structured mentorship for early-career nurses, equitable workload distribution, and gender-sensitive well-being programs to strengthen long-term workforce resilience.

To provide a clearer overview of the reported prevalence of burnout among nurses during the COVID-19 pandemic, the main findings from systematic reviews, meta-analyses, and large-scale surveys are summarized in Table 2. These data highlight variations in prevalence across specialties, regions, and sociodemographic groups, while also illustrating the methodological diversity of the included studies.

### 3.3. Contributing Factors

Burnout among nurses during the COVID-19 pandemic was not the result of a single stressor, but rather the accumulation of multiple, interacting pressures at both the individual and organizational levels. Among the most consistently identified drivers were excessive workload, prolonged shifts, and rapidly evolving clinical protocols, all of which placed unprecedented strain on frontline staff [15]. Limited access to personal protective equipment (PPE) in the early phases of the pandemic further intensified emotional exhaustion, as fear of infection and concern about transmitting the virus to family members became pervasive sources of distress [3,23].

Social and professional isolation also played a significant role. Nurses frequently reported being stigmatized within their communities, perceived as potential carriers of infection, which compounded their stress and undermined available support networks [3]. Ethical dilemmas, including rationing of scarce resources or delaying elective but necessary procedures, added another layer of moral and psychological burden, often overlapping with experiences of moral injury [4].

Demographic factors have been shown to modulate vulnerability. Younger nurses, those with fewer years of professional experience, and female nurses were disproportionately affected, reporting higher levels of emotional exhaustion and lower levels of perceived support compared with their older or more experienced colleagues [5,11]. These findings suggest that burnout during COVID-19 was shaped not only by structural and organizational deficiencies but also by individual vulnerabilities, which together created a high-risk environment for sustained psychological strain.

Overall, existing studies provide strong and consistent evidence linking workload, staffing deficits, and ethical strain to burnout, demonstrating global prevalence above 30% [6]. However, most analyses remain cross-sectional, limiting causal interpretation. Longitudinal designs exploring mediators such as organizational culture and resilience are still scarce.

## 4. Moral Injury in Nursing During COVID-19

Moral injury (MI) in healthcare refers to the psychological, existential, and relational harm that occurs when clinicians perceive, perpetrate, witness, or fail to prevent actions that violate their core moral values in the course of care. In nursing, MI was catalyzed by pandemic realities such as rationing, enforced visitor restrictions, inconsistent policies, and perceived betrayals by systems or authorities—phenomena distinct from ordinary occupational stress. Conceptual work in medicine emphasizes that MI is not a personal weakness but a system-linked insult that requires organizational recognition and repair strategies [24,25].

### 4.1. Conceptual Distinction from Moral Distress

Recent scholarship emphasizes that moral distress (MD) and moral injury (MI) should be viewed along a continuum of moral harm rather than as separate constructs. MD reflects acute psychological discomfort caused by ethical constraints in clinical work, while repeated or unresolved exposures may evolve into MI—a deeper and more enduring moral wound marked by loss of trust and professional identity [26,27]. The concept of potentially morally injurious events (PMIEs) captures this transition, describing situations where nurses act, fail to act, or witness actions that violate their moral values [28].

Although both phenomena share ethical origins, they differ in duration, psychological impact, and organizational consequences. MD typically resolves when institutional barriers are removed, whereas MI often persists without targeted intervention and is associated with outcomes such as PTSD, depression, and suicidality [29,30,31,32,33,34].

To provide a structured overview of evidence-based strategies, Table 3 summarizes organizational and systemic measures recommended by the National Academy of Medicine (NAM) in its perspective paper “A Path to Improved Health Care Worker Well-Being: Lessons from the COVID-19 Pandemic.” [13]. These recommendations emphasize the importance of recognizing and valuing the healthcare workforce, reducing administrative burden, expanding access to mental health services, and implementing sustainable workforce policies. By adopting such measures, healthcare systems can strengthen resilience, mitigate burnout and moral injury, and ensure the delivery of safe and high-quality patient care.

As illustrated in Table 3, MD and MI differ not only in their definitions and triggers but also in their long-term psychological and organizational consequences, requiring distinct intervention strategies. Building on this conceptual distinction, the following section examines the prevalence and severity of MI among nurses during the COVID-19 pandemic, highlighting the extent of the phenomenon and its implications for workforce resilience.

### 4.2. Measurement in Nursing and Healthcare Settings

Accurate measurement of moral injury in nursing and healthcare settings is essential for both research and practice, enabling not only prevalence estimation but also the design, monitoring, and evaluation of organizational interventions. Two validated instruments are currently most widely used to quantify MI in healthcare workers, including nurses: the Moral Injury Symptoms Scale–Health Professional (MISS-HP) and adaptations of the Moral Injury Events Scale to healthcare contexts (e.g., MIES-HC) [35].

The MISS-HP has demonstrated acceptable reliability and construct validity across diverse samples of healthcare workers. Validation among Chinese health professionals during the COVID-19 pandemic confirmed its strong psychometric properties, with nurse subsamples consistently showing robust internal consistency and factorial validity [36].

Similarly, the MIES-HC—an adaptation of the original Moral Injury Events Scale from military psychology—has been applied successfully to healthcare contexts. Studies show that this tool captures exposure to potentially morally injurious events (PMIEs), such as rationing of care or institutional betrayal, and correlates strongly with downstream psychological outcomes, including depression, PTSD, and turnover intent [32].

From a practical perspective, these instruments are more than diagnostic tools: they enable screening and benchmarking of MI prevalence across institutions, provide metrics for cross-national comparisons, and serve as outcome measures for moral repair programs. Their integration into routine workforce well-being monitoring could be further enhanced by digital health platforms, enabling real-time tracking, early identification of high-risk staff, and timely deployment of resilience-building interventions [13].

To better understand how moral injury (MI) is assessed in healthcare settings, particularly in nursing, two main instruments have been validated and applied in recent years. The Moral Injury Symptoms Scale–Health Professional (MISS-HP) focuses on the subjective experience of MI symptoms such as guilt, shame, and loss of meaning, while the Moral Injury Events Scale adapted to healthcare (MIES-HC) measures exposure to potentially morally injurious events (PMIEs), including perceived transgressions and betrayals. Table 4 provides a comparative overview of these instruments, summarizing their scope, psychometric validation, strengths, and practical applications in both clinical and organizational contexts.

Taken together, MISS-HP and MIES-HC represent validated, feasible, and scalable approaches to quantifying MI in nurses. By embedding these instruments within organizational health strategies, healthcare leaders can move beyond anecdotal recognition of moral harm to evidence-based planning of leadership accountability mechanisms, targeted psychosocial support, and technology-enabled resilience interventions.

### 4.3. Prevalence and Severity During the Pandemic

Emerging data consistently indicate that moral injury (MI) became a pervasive occupational hazard for nurses during the COVID-19 pandemic. Large-scale studies using validated instruments such as MISS-HP and MIES-HC showed that a substantial proportion of nurses reported moderate to severe symptom burdens, particularly in intensive care units, emergency departments, and long-term care facilities [32,39]. In contrast, those in outpatient or community-based services reported lower, yet still clinically relevant, scores, confirming that MI affected all levels of nursing practice to varying degrees [40].

Longitudinal evidence further demonstrates that moral strain persisted over time; multi-wave studies spanning 2020–2022 reported sustained MI symptoms even as pandemic pressures eased [27,41,42]. This persistence distinguishes MI from transient distress and underscores its potential to disrupt professional identity and long-term workforce stability.

Both quantitative and qualitative findings converge in highlighting the multifaceted impact of MI. Quantitative studies link higher MI scores with PTSD, depression, anxiety, and suicidal ideation, emphasizing its dual role as a clinical and organizational threat [26,38]. Qualitative analyses complement these results, showing how repeated potentially morally injurious events (PMIEs)—such as constrained care, institutional betrayal, and leadership failures—produce enduring moral wounds and erode trust in healthcare institutions [43,44,45].

To facilitate quick comparison without redundancy, Table 5 summarizes reported prevalence ranges of moral injury among nurses across care settings.

These comparative ranges show the highest MI burden in acute, high-intensity environments, while remaining clinically relevant across all settings, reinforcing the need for system-level mitigation strategies.

**Figure 1:**
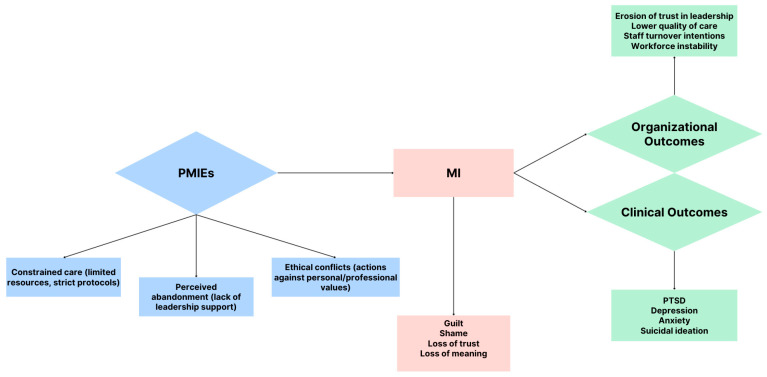
Conceptual pathway from potentially morally injurious events (PMIEs) to moral injury (MI) and its consequences. Exposure to constrained care, perceived abandonment, and ethical conflicts increases the risk of MI, characterized by guilt, shame, loss of trust, and loss of meaning. These symptoms lead to both clinical outcomes and organizational outcomes. Abbreviations: PMIEs—Potentially Morally Injurious Events; MI—Moral Injury; PTSD—Post-Traumatic Stress Disorder.

To illustrate the relationship between exposure, symptomatology, and outcomes, Figure 1 presents a conceptual model connecting PMIEs to the development and consequences of MI. As shown, systemic stressors like constrained care and ethical conflicts can lead to guilt, shame, loss of meaning, and trust, which in turn result in both psychological sequelae (e.g., PTSD, depression) and organizational repercussions (e.g., decreased care quality, turnover intentions).

Collectively, these findings confirm that MI prevalence and severity were both substantial and enduring during the pandemic, reinforcing the urgent need for organizational strategies focused on moral repair, resilience building, and preventive frameworks to protect the nursing workforce in future crises.

Together, these findings highlight the systemic and enduring nature of MI. However, it is important to note that most studies included were cross-sectional, limiting causal inference. Longitudinal and interventional data remain scarce, underscoring the need for future research to clarify temporal relationships and evaluate the long-term effectiveness of organizational interventions.

### 4.4. Clinical Consequences of Moral Injury

Moral injury (MI) exerts profound clinical effects on nurses, extending beyond transient distress and shaping long-term trajectories of mental health and professional functioning. Recent studies have consistently shown strong associations between MI severity and the onset of psychiatric disorders, particularly PTSD, depression, and anxiety disorders [31]. Unlike moral distress, which may diminish once institutional barriers are addressed, MI often leads to enduring psychological impairment, including intrusive memories, guilt, and shame, which compromise recovery even after the acute crisis has passed [46,47].

A growing body of evidence links MI to suicidal ideation and behavior among healthcare professionals. Network analyses have identified “betrayal” and “loss of trust” as central bridge symptoms connecting MI to suicidality. Such findings underscore the necessity of viewing MI not only as an occupational stressor but as a major mental health risk requiring systematic screening and follow-up [30,48,49].

Clinical consequences are not limited to psychiatric morbidity. Nurses with elevated MI scores also report somatic complaints, disrupted sleep, and emotional numbing, which in turn affect work performance and interpersonal relationships. These symptoms contribute to functional decline, impairing both personal well-being and the ability to provide safe, empathetic patient care [50,51,52].

Longitudinal findings further emphasize the chronic nature of MI. Unlike acute stress reactions, MI-related symptoms demonstrate little spontaneous remission over time, persisting well beyond the resolution of pandemic-related crises [5,53,54]. This persistence highlights the need for structured interventions, such as targeted psychotherapy, resilience training, and organizational programs for moral repair, to mitigate the clinical burden and prevent attrition from the nursing workforce.

### 4.5. Organizational Consequences

MI not only affects the mental health of individual nurses, but also undermines the organizational fabric of healthcare systems. Nurses who experience sustained MI frequently report erosion of trust in leadership and institutions, perceiving organizational betrayal as a core driver of their distress. This breakdown in relational trust reduces adherence to institutional goals and weakens engagement in quality improvement processes, ultimately compromising organizational resilience [34,55,56].

A growing body of evidence indicates that MI contributes significantly to workforce instability. Nurses experiencing MI are more prone to absenteeism, presenteeism, and an increased intention to leave the profession, exacerbating the already critical shortage in the post-pandemic period [57]. A 2023 systematic review demonstrated that exposure to potentially morally injurious events (PMIEs) was strongly associated with intention to leave the profession and decreased job satisfaction, particularly in high-stress settings such as intensive care and emergency departments [21].

MI also undermines team functioning and interprofessional collaboration. Feelings of betrayal or injustice foster cynicism and withdrawal, which erode communication and cooperation across care teams. Qualitative studies among nurses describe a shift from a collaborative ethos to defensive practices, driven by fear of further moral compromise [58,59]. This dynamic not only weakens team cohesion but also contributes to lower quality of care and increased medical errors, as disengaged staff are less likely to advocate for patients or report safety concerns.

Finally, the financial consequences of MI are substantial. Staff turnover, sick leave, and decreased productivity impose heavy costs on healthcare organizations, which are compounded by reduced patient satisfaction and reputational risks [60,61]. These systemic outcomes highlight that MI should not be viewed solely as a clinical issue but as a strategic organizational threat requiring leadership accountability and institutional moral repair programs.

### 4.6. Interventions and Innovative Strategies

Efforts to mitigate burnout and MI among nurses during and after the COVID-19 pandemic increasingly emphasize system-level, innovative interventions rather than individual coping alone. Recent evidence highlights that sustainable solutions must combine organizational reform, supportive leadership, and digital innovation to foster resilience and retain the nursing workforce [34,62].

The conceptual underpinnings of these interventions can be interpreted through the Job Demands–Resources (JD–R) model, which provides a theoretical lens to explain how organizational and psychological factors interact to influence nurse well-being and resilience [63]. According to this framework, job demands—such as high workload, ethical strain, and emotional labor—exert chronic pressure that depletes energy and emotional resources, predisposing individuals to burnout and moral injury [64]. Conversely, job resources—including supportive leadership, autonomy, adequate staffing, and access to digital tools—act as protective mechanisms that buffer the impact of demands, fostering engagement and moral resilience [63]. Applying the JD–R perspective clarifies that burnout and moral injury are not solely personal or clinical outcomes, but predictable responses to sustained organizational imbalance between demands and resources. Therefore, organizational innovation should prioritize strategies that systematically strengthen resource availability and redistribute demands to sustain psychological well-being and ethical integrity across nursing teams.

Flexible staffing models, workload redistribution, and protected recovery time have shown measurable benefits in mitigating moral distress and burnout among nurses. Pilot evaluations of workload-equity interventions reported reductions in perceived burnout risk by approximately 20–25% and increases in nurse satisfaction exceeding 25%, following implementation of acuity-based assignment tools and flexible scheduling programs [65,66,67]. These findings substantiate that workload optimization contributes to psychological safety and resilience, preventing escalation from moral distress to moral injury.

Supportive and ethical leadership. Nursing leadership that prioritizes transparency, open communication, and acknowledgment of staff suffering has been shown to mitigate perceptions of betrayal—a core trigger of moral injury (MI). Recent empirical studies demonstrate that authentic and supportive leadership significantly enhances moral resilience and reduces the risk of MI among nurses and other high-risk professionals [68,69]. Interventions such as structured moral repair dialogs and “ethics rounds” provide nurses with a platform to process potentially morally injurious events (PMIEs) collectively, fostering a culture of psychological safety and shared accountability [70,71].

Supportive and ethical leadership. Nursing leadership that prioritizes transparency, open communication, and acknowledgment of staff suffering has been shown to mitigate perceptions of betrayal—a core trigger of MI. Interventions such as structured moral repair dialogs and “ethics rounds” give nurses a platform to process PMIEs collectively, fostering a culture of psychological safety [72,73].

Post-pandemic, technology has become central to innovative care models aimed at preventing burnout and moral injury. Mobile applications delivering just-in-time psychological support (e.g., mindfulness and coping apps) have demonstrated reductions of 15–25% in stress and burnout scores among nurses participating in 4–8-week interventions [72]. AI-driven scheduling systems and digital dashboards for workload prediction improved fairness perceptions and decreased moral distress levels in high-acuity wards [73]. Tele-mentoring and peer-support platforms, such as virtual “resilience circles,” have also been shown to improve emotional well-being and lower MI indicators by 20–30% in pilot programs implemented across U.S. and European hospitals [72,73]. Collectively, these findings underscore that integrating scalable digital tools with existing psychosocial support frameworks can enhance accessibility, early intervention, and sustainability of workforce resilience.

Interprofessional collaboration and peer-support networks. Structured peer-support programs—such as “buddy systems” and resilience coaching—help normalize emotional challenges and reduce stigma around moral suffering. These models strengthen team cohesion, countering the fragmentation of collaboration observed during the pandemic [74].

Countries that embedded national nurse well-being strategies—such as mandatory safe staffing ratios, funding for resilience programs, and protected leave—reported lower turnover rates post-COVID-19 compared to systems without such policies [75]. Comparative analyses underscore that policy innovation is essential to address MI as a systemic occupational hazard rather than an individual failing [76,77]. In California, multi-study syntheses reported lower burnout, reduced job dissatisfaction, and decreased intentions to leave among nurses compared with states without mandated ratios [78]. International reports (ICN, OECD) further highlight that national well-being strategies—including safe staffing policies, investment in working conditions, and protected rest/leave—are key levers for recruitment and retention in the post-pandemic period [52,79,80].

However, the large-scale implementation of such measures remains constrained by limited financial resources, unequal policy adoption across regions, and organizational resistance to restructuring long-standing staffing models. In many healthcare systems, especially those facing chronic workforce shortages, these constraints have delayed or diluted the intended benefits of policy reforms. Acknowledging these limitations is essential to realistically assessing both the feasibility and the sustainability of systemic interventions aimed at mitigating burnout and moral injury.

Research consistently demonstrates the organizational toll of moral injury—lower trust, turnover, and financial costs—but controlled evaluations of institutional repair or prevention programs are still rare. Strengthening methodological rigor and measuring long-term recovery remain major priorities.

### 4.7. Future Directions and Research Gaps

Given the secondary nature of the data synthesized in this narrative meta-synthesis, future research priorities should be weighted more heavily than policy recommendations. Accordingly, we outline below the key directions needed to consolidate the field.

Although the literature on burnout and moral injury among healthcare workers has expanded significantly during and after the COVID-19 pandemic, important knowledge gaps remain. Most available studies are cross-sectional, which limits the ability to draw causal inferences about the long-term effects of moral injury and burnout on mental health and professional outcomes [39,41]. There is a clear need for longitudinal and prospective cohort studies to track the trajectory of distress, recovery, and resilience over time.

Another gap concerns the limited evidence on intervention strategies. While organizational interventions such as staffing policies, leadership approaches, and telemedicine solutions have been proposed to mitigate burnout, their effectiveness remains underexplored in controlled trials [34,60,72]. Moreover, the literature highlights that structural determinants such as nurse-to-patient ratios, rationing of care, and turnover directly influence both staff well-being and patient safety, yet evidence on how policy changes could reduce moral injury is still scarce [67,79,80].

In addition, the diversity of healthcare workers has not been sufficiently addressed. Few studies explicitly explore how gender, ethnicity, and socio-economic context affect vulnerability to moral distress and burnout [44,70]. Similarly, while validated measurement tools such as the Moral Injury Symptom Scale have been adapted cross-culturally, further psychometric validation in different cultural and healthcare settings is required [35,38].

Finally, extreme outcomes such as suicidal ideation, suicidal behavior, and workforce attrition have only recently been systematically documented, and more research is needed to design preventive strategies [46,48]. Addressing these gaps will be crucial for developing evidence-based policies and interventions to safeguard the well-being of healthcare professionals and ensure sustainable healthcare systems.

Achievements to date. The field has progressed by (i) establishing distinct conceptual boundaries between burnout, moral distress, and moral injury; (ii) documenting high yet heterogeneous prevalence across settings (ICU, ED, LTC, acute care); (iii) adapting and validating measurement tools for moral injury in healthcare (e.g., MISS-HP, MIES-HC); and (iv) piloting organization-level strategies (staffing optimization, ethical leadership training, digital workload-reduction), with early signals of reduced burnout and improved perceived safety culture [37].

Remaining methodological and theoretical gaps. Despite these achievements, research remains methodologically fragmented. Most studies are cross-sectional, rely on self-report instruments, and use heterogeneous moral injury scales, which hinder comparability and synthesis. Although this review has applied the Job Demands–Resources (JD–R) model to explain the mechanisms linking organizational factors with burnout and moral injury, further theoretical refinement is needed to develop integrative models specifically tailored to moral injury in nursing contexts. Few studies test mediators or moderators, such as organizational ethics climate, leadership style, or team cohesion [81].

Future research should therefore prioritize standardized instruments for moral injury, longitudinal and mixed-methods designs, and intervention trials examining how organizational factors—like acuity-based staffing, ethics-focused leadership training, and digital workload optimization—affect both nurse resilience and patient outcomes.

In summary, several key research gaps remain to be addressed. Future studies should:

(1) Establish causal and temporal pathways linking burnout, moral distress, and moral injury using longitudinal or prospective cohort designs;
(2) Conduct controlled intervention trials testing organizational and policy-level strategies (staffing optimization, ethical leadership, digital support tools);
(3) Enhance cross-cultural and psychometric validation of moral injury measurement scales;
(4) Investigate sociodemographic moderators (gender, age, professional seniority, cultural context) influencing vulnerability and recovery; and
(5) Explore severe downstream outcomes such as suicidal ideation, turnover, and chronic psychiatric sequelae to develop early prevention frameworks.

Articulating and addressing these specific priorities will help move the field from descriptive to mechanistic and interventional research, consolidating an evidence-based foundation for future policy and practice.

Research should prioritize longitudinal and controlled trials that quantify effects of acuity-based staffing, ethical leadership training, and digital workload optimization on burnout and retention. Policy work should advance safe staffing standards, funding mechanisms for well-being infrastructures, and accountability for institutional ethics climates. At the practice level, organizations should embed ethics rounds/moral repair dialogs, mentorship for early-career nurses, and data-informed scheduling, integrating continuous monitoring to sustain gains.

## 5. Policy and Practice Implications

The COVID-19 pandemic underscored that both burnout and moral injury among nurses are not isolated phenomena but systemic hazards that demand policy-level solutions. Post-pandemic recovery requires moving beyond individual coping strategies toward structural reforms that foster organizational resilience and sustainability in the nursing workforce. Several domains of policy and practice have emerged as priorities.

### 5.1. Safe Staffing and Workload Equity

Legislative frameworks introducing safe nurse-to-patient ratios have been shown to reduce burnout and turnover while improving patient outcomes. Evidence from California and Queensland indicates that mandated ratios enhance nurse well-being and job satisfaction, particularly in high-acuity settings, while reducing mortality and adverse events [71,73]. These policies demonstrate that equitable workload distribution is not only a workforce issue but also a patient safety imperative.

### 5.2. Professional Autonomy and Ethical Climate

Institutional policies that strengthen professional autonomy—such as shared governance models, participatory decision-making, and protected time for ethical reflection—support nurses in regaining control over morally challenging contexts. By formally embedding ethics rounds or moral deliberation forums, organizations can reduce the escalation of moral distress into moral injury [82].

### 5.3. Leadership Development and Accountability

Supportive and ethical leadership has emerged as a cornerstone of resilience. Policies must incentivize leadership training focused on transparent communication, psychological safety, and accountability for workplace culture. Leaders who acknowledge the moral weight of frontline decisions foster trust and mitigate the sense of institutional betrayal that characterizes moral injury [83,84].

### 5.4. Digital and Technological Innovations

National and institutional strategies should leverage technology to support nurse well-being. Digital staffing tools that integrate acuity-based assignment systems have been piloted with promising results in workload fairness [85]. Similarly, mobile applications providing psychological first aid, tele-mentoring, and peer-support platforms have shown potential to address acute stress and moral distress in real time [86].

### 5.5. Integrated Mental Health and Well-Being Programs

Policies should move toward embedding comprehensive well-being programs within health systems. Protected mental health leave, resilience training, and access to counseling must be institutionalized as rights, not optional benefits. International analyses from the International Council of Nurses (ICN, 2023) emphasize that sustained investment in workforce well-being is key to retention and recruitment in the post-pandemic era [53,87].

### 5.6. Global and Comparative Perspectives

Cross-national comparisons suggest notable variation in how countries protected nurses during COVID-19. For example, Korea has progressively adopted staffing policy reforms with financial incentives tied to nurse-to-patient ratios, influencing hospital staffing behavior [88]. Additionally, Wales’s Nurse Staffing Levels Act 2016 mandates planning and reporting of nurse staffing levels and includes retention measures [80]. While direct cross-country empirical comparisons are scarce, these policy-level strategies reflect a shift toward systemic support of the nursing workforce and hint at associations with lower attrition when such infrastructures are in place [78,89,90].

## 6. Conclusions

The COVID-19 pandemic has exposed the deep vulnerability of the nursing workforce to burnout and moral injury, conditions that reflect structural—not individual—failures within healthcare systems. Burnout, characterized by emotional exhaustion and depersonalization, and moral injury, rooted in ethical conflict and institutional betrayal, have both intensified under systemic strain. These phenomena underscore that the pandemic amplified pre-existing weaknesses in organizational culture, leadership, and staffing structures. While most of the reviewed evidence was gathered during the pandemic’s peak, the persistence of burnout and moral injury into the recovery phase illustrates that these are now enduring occupational hazards that define the post-pandemic era.

Sustainable recovery requires a paradigm shift: healthcare institutions must be re-envisioned as moral communities grounded in trust, fairness, and psychological safety. Multi-level strategies—combining safe staffing, ethical and transparent leadership, and digital well-being tools—are essential to rebuild resilience and restore meaning in professional practice.

Future efforts should move beyond description toward interventional and longitudinal research, identifying evidence-based pathways for moral repair and workforce sustainability.

Addressing moral injury through ethical leadership, staffing reform, and system-level accountability is crucial to sustaining the global nursing workforce in the post-pandemic era. These priorities align with international frameworks from the World Health Organization (WHO) and the International Council of Nurses (ICN), reinforcing the moral and policy imperative to protect those who care for others.

## Figures and Tables

**Table 1:** Summary of burnout prevalence among nurses by region and specialty (2020–2023). Data synthesized from meta-analyses and large-scale surveys. ICU—Intensive Care Unit; PPE—Personal Protective Equipment; HCW—Healthcare Worker.

Region/Specialty	Estimated Burnout Prevalence	Key Moderators/Notes
Global pooled estimate [3,14,22]	~30–50%	Persistent above pre-pandemic levels
Critical care/ICU nurses [14,22]	~45–55%	Highest exposure to acutely ill patients, ethical dilemmas
Emergency departments [22]	~43%	Highest risk group among healthcare workers
Oncology nurses [22]	~40–45%	High emotional load and sustained exposure to suffering
Outpatient/community care [22]	~20–30%	Lower but clinically relevant burnout levels
North America [21]	~30% of HCWs considering leaving profession	Burnout and poor working conditions main drivers
Regions with staffing shortages/limited PPE [21]	>50%	Lack of institutional support and protection
Regions with adequate ratios and support programs [21]	<30%	Early intervention, protective organizational measures

**Table 2:** Prevalence of burnout among nurses during the COVID-19 pandemic according to systematic reviews, meta-analyses, and large-scale surveys (2020–2023). The table summarizes prevalence estimates, study contexts, and key subgroup findings. Abbreviations: MBI—Maslach Burnout Inventory; HCWs—Healthcare Workers; COVID-19-designated wards—hospital units assigned exclusively for COVID-19 patient care.

Study Type and Year	Population/Context	Key Findings (Burnout Prevalence)	Notes/Subdimensions
Systematic review and meta-analysis [3]	Global nursing workforce	Emotional exhaustion: 34.1%; Depersonalization: 12.6%; Low accomplishment: 15.2%	Based on Maslach Burnout Inventory (MBI)
Systematic review [14,22]	Nurses, multi-country	30–50% of nurses reported clinically significant burnout	Variation by specialty and pandemic phase
Meta-analysis [22]	Emergency department HCWs (incl. nurses)	Overall prevalence: ~43%	Nurses among the most affected subgroups
Cross-sectional surveys [14]	Critical care & COVID-19-designated wards	Burnout prevalence at the higher end of global range (>40%)	Driven by workload, PPE shortages, ethical dilemmas
Mixed-methods study (USA, 2023) [21]	Healthcare workers (incl. nurses)	~30% considered leaving the profession	Burnout and deteriorating working conditions as drivers
Large-scale surveys [14,21]	Younger nurses (<5 years exp.)	Higher risk of severe burnout vs. senior nurses	Reflects lower coping resources
Multi-country comparative analyses [3]	Male vs. female nurses	Women: higher emotional exhaustion; Men: higher depersonalization	Gender effects inconsistent across studies

**Table 3:** Comparison between moral distress (MD) and moral injury (MI) in nursing practice, with emphasis on definition, triggers, duration, psychological consequences, organizational outcomes, and intervention strategies, supported by recent empirical and conceptual studies.

Dimension	MD	MI
Definition	Psychological discomfort when nurses know the ethically right action but are unable to act due to constraints [3]	Deep, enduring psychological harm caused by acting against, or witnessing violations of, core moral values [2]
Primary Triggers	Institutional barriers, resource limitations, conflicting protocols [30]	Potentially morally injurious events (PMIEs) such as rationing care, witnessing preventable suffering, or perceived betrayal by leadership [31]
Duration	Typically, acute or episodic, may resolve if barriers are removed [11]	Persistent and cumulative, often long-lasting without targeted intervention [32]
Psychological Impact	Frustration, guilt, anxiety, emotional exhaustion [17]	PTSD, depression, suicidality, erosion of professional identity [33]
Organizational Consequences	Decreased job satisfaction, increased turnover intentions [21]	Workforce attrition, impaired trust in institutions, long-term organizational harm [5]
Intervention Strategies	Ethics consultations, workflow redesign, improved communication, peer support [34]	Moral repair through acknowledgment of harm, leadership accountability, institutional reforms, resilience-building interventions [13]

**Table 4:** Comparative characteristics of validated instruments for assessing moral injury (MI) in healthcare workers, with a focus on nursing practice. The table summarizes the scope, validation evidence, strengths, and practical applications of the Moral Injury Symptoms Scale–Health Professional (MISS-HP) and the Moral Injury Events Scale adapted to healthcare (MIES-HC). Abbreviations: MI—Moral Injury; MISS-HP—Moral Injury Symptoms Scale–Health Professional; MIES-HC—Moral Injury Events Scale–Healthcare version; PMIEs—Potentially Morally Injurious Events; HCWs—Healthcare Workers.

Instrument	Scope and Structure	Validation Evidence	Strengths	Practical Applications
MISS-HP	10-item scale assessing MI symptoms (guilt, shame, loss of meaning, betrayal, trust)	Internal reliability = 0.75; PCA + CFA explain ~56.8% variance; convergent validity with clinician burnout (r = 0.57); discriminant validity with depression/anxiety (r = 0.25–0.37); ROC cut-off (≥36) 84% sensitivity/93% specificity [37]	Tailored to health professionals, moderate length, psychometrically supported	Screening MI symptoms, benchmarking, evaluating moral repair interventions
MIES adapted to healthcare (e.g., Italian version)	MIES version adapted to health settings measuring PMIE exposure (transgressions, betrayals)	Validation in Italian HCWs (n = 794, ~46% nurses): three-factor structure (self-transgressions, other-transgressions, betrayals); convergence with PTSD, anxiety, depression, burnout [38]	Captures systemic and event-level triggers; able to distinguish exposure types	Identifying organizational triggers, correlating PMIEs with outcomes, guiding preventive structures

**Table 5:** Reported prevalence ranges of moral injury (MI) among nurses by care setting during the COVID-19 pandemic. ICU—Intensive Care Unit; ED—Emergency Department; LTC—Long-Term Care.

Care Setting	MI Prevalence (Range)	Key Contributing Factors
ICU/Critical Care [32,40,43]	40–55%	Triage dilemmas, high mortality exposure, resource scarcity
ED [40,43]	35–50%	Crisis triage, sustained acute exposure
LTC [2,42]	30–45%	Perceived abandonment, staffing gaps
Outpatient/Community [41]	15–25%	Lower acute exposure; under-support

## Data Availability

No new data were created or analyzed in this study. Data sharing is not applicable to this article.
